# BACE1 Is Necessary for Experience-Dependent Homeostatic Synaptic Plasticity in Visual Cortex

**DOI:** 10.1155/2014/128631

**Published:** 2014-05-14

**Authors:** Emily Petrus, Hey-Kyoung Lee

**Affiliations:** Zanvyl-Krieger Mind/Brain Institute, Solomon H. Snyder Department of Neuroscience, Johns Hopkins University, 3400 N. Charles Street, Dunning Hall 348, Baltimore, MD 21218, USA

## Abstract

Alzheimer's disease (AD) is the most common form of age-related dementia, which is thought to result from overproduction and/or reduced clearance of amyloid-beta (A**β**) peptides. Studies over the past few decades suggest that A**β** is produced in an activity-dependent manner and has physiological relevance to normal brain functions. Similarly, physiological functions for **β**- and **γ**-secretases, the two key enzymes that produce A**β** by sequentially processing the amyloid precursor protein (APP), have been discovered over recent years. In particular, activity-dependent production of A**β** has been suggested to play a role in homeostatic regulation of excitatory synaptic function. There is accumulating evidence that activity-dependent immediate early gene Arc is an activity “sensor,” which acts upstream of A**β** production and triggers AMPA receptor endocytosis to homeostatically downregulate the strength of excitatory synaptic transmission. We previously reported that Arc is critical for sensory experience-dependent homeostatic reduction of excitatory synaptic transmission in the superficial layers of visual cortex. Here we demonstrate that mice lacking the major neuronal **β**-secretase, BACE1, exhibit a similar phenotype: stronger basal excitatory synaptic transmission and failure to adapt to changes in visual experience. Our results indicate that BACE1 plays an essential role in sensory experience-dependent homeostatic synaptic plasticity in the neocortex.

## 1. Introduction

Alzheimer's disease (AD) is a progressive neurodegenerative disease, which is thought to initiate by accumulation of A*β* peptide and synaptic dysfunctions [1]. AD is primarily characterized by impairment in memory formation, but the disease accompanies failure in sensory processing as well as cognitive abilities. Impairment in visual processing is sometimes observed in AD patients [2–6] and in particular severe deficits in visuospatial function are observed in a subpopulation of AD with Bálint's syndrome [7–9]. While primary sensory cortices are thought to be less affected by AD [3, 10, 11], A*β* plaques and neurofibrillary tangle—hallmarks of AD—are present in the superficial layers of primary visual cortex (V1) from postmortem AD patients [7, 12, 13] and mouse models of AD [14, 15]. In a recent study using* in vivo* 2 photon Ca^2+^ imaging in V1 of a mouse model of AD (APP23xPS45), an age-dependent progressive loss of neuronal orientation tuning paralleled the increase in A*β* load [14]. The orientation tuning defects were limited to neurons with hyperactivity under basal conditions, which are often found in close proximity to A*β* plaques [16]. Furthermore, the loss of orientation tuning accompanied a progressive deficit in a visual pattern discrimination task [14], which suggests that the neuronal dysfunction may lead to functional decline in visual processing. In addition, ocular dominance plasticity in V1 is impaired in a different mouse model of AD (APPswe; PS1ΔE9) at an early developmental age before excessive A*β* accumulation is observed [17], which suggests that APP and/or moderate production of A*β* may negatively impact sensory cortex plasticity.

Recently manipulations that prevent A*β* production have revealed a crucial role of A*β* in normal synaptic function (reviewed in [18]), particularly homeostatic processes that provide stability to neuronal activity [19–21]. A*β* is produced in an activity-dependent manner by sequential cleavage of the amyloid precursor protein (APP) by *β*- and *γ*-secretases [20, 22], which happens mainly in endosomes [23–25]. In hippocampal neurons, overproduction or exogenous application of A*β* can induce endocytosis of AMPA receptors to reduce synaptic drive [19, 20, 26]. Notably, the activity dependence of A*β* production requires the immediate early gene Arc [24], which is critical for activity-dependent endocytosis of AMPA receptors [27] and homeostatic synaptic scaling in both hippocampus [28] and V1 [29]. In V1, a few days of visual deprivation homeostatically scale up the strength of excitatory synapses [29–34], which is observed during the critical period through adulthood at least in the superficial layers [35]. This process is readily reversed by a few hours of visual experience [29, 30, 33, 35]. Bidirectional homeostatic synaptic plasticity induced by changes in visual experience recruits distinct molecular signalings, which are not exactly the inverse of each other. For example, upscaling induced by losing vision requires phosphorylation of AMPA receptors and synaptic expression of Ca^2+^-permeable AMPA receptors [30, 33], while downscaling by visual experience depends on Arc [29]. Despite the wealth of knowledge suggesting that visual cortex is an excellent system to study experience-dependent homeostatic synaptic plasticity (reviewed in [36]), a direct test of whether A*β* plays a role in this process is lacking. To address this, we used a knockout (KO) mouse, which lacks the major neuronal *β*-secretase (BACE1) and hence A*β* [37, 38] (but see [39]). We found that BACE1 KOs exhibit abnormally enhanced basal excitatory synaptic transmission in layer 2/3 (L2/3) pyramidal neurons of V1 and lack sensory experience-dependent homeostatic synaptic plasticity. Our results suggest a novel role of BACE1 in homeostatic regulation of excitatory synaptic transmission in V1.

## 2. Materials and Methods

### 2.1. Animals

Male mice were derived from heterozygous breeders and identified as BACE1^−/−^ (KO) or BACE1^+/+^ (WT) via polymerase chain reaction (PCR) analysis as described previously [38]. Mice were raised in 12-hour light/12-hour dark cycle until postnatal days 22–26 (p22–p26), at which point some mice were dark-exposed (DE) for 2 days while others remained in a normal lighted environment (normal reared, NR). DE animals were cared for using infrared vision goggles with dim infrared light. Some DE mice were returned to normal light conditions for 2 hours to study the effect of light reexposure (LE). All experiments were approved by the Johns Hopkins University Institutional Animal Care and Use Committee (IACUC) and followed the guidelines of the Animal Welfare Act.

### 2.2. Visual Cortex Slice Preparation

Mice between p24 and p28 were deeply anesthetized with isofluorane vapors in a chamber placed inside a chemical fume hood. The chamber was light tight for DE experimental groups. After absence of corneal reflex, mice were decapitated and the brain was isolated in ice-cold dissection buffer (in mM: 212.7 sucrose, 5 KCl, 1.25 NaH_2_PO_4_, 26 NaHCO_3_, 10 glucose, 3 MgCl_2_, and 1 CaCl_2_), which was bubbled with 95% O_2_/5% CO_2_ gas. Primary visual cortex blocks were rapidly dissected and coronally sectioned into 300 *μ*m thick slices using a Vibratome 3000 plus microslicer (Ted Pella). Slices were gently transferred to a submersion-type holding chamber filled with artificial cerebral spinal fluid (ACSF, in mM: 125 NaCl, 5 KCl, 1.25 NaH_2_PO_4_, 26 NaHCO_3_, 10 glucose, 1 MgCl_2_, and 2 CaCl_2_), which was saturated with 95% O_2_/5% CO_2_. The slices were allowed to recover at room temperature for 1 hour before recording.

### 2.3. Electrophysiology

For mEPSC recording, slices were transferred to a submersion-type recording chamber mounted on the fixed stage of an upright microscope (E600 FN; Nikon, Tokyo, Japan) with oblique infrared (IR) illumination. AMPA receptor-mediated miniature excitatory postsynaptic currents were isolated pharmacologically with 1 *μ*M tetrodotoxin (TTX), 20 *μ*M bicuculline, and 100 *μ*M DL-2-amino-5 phosphonopentanoic acid (DL-APV). These agents were added to ACSF (bubbled with 95% O_2_/5% CO_2_ and maintained at 30 ± 1°C), which was continually perfused at a rate of 2 mL/min. Cells in layers 2/3 of primary visual cortex were identified by their pyramidal-shaped soma and apical dendrite pointing towards the pia. Pyramidal neurons were patched using a whole-cell patch pipette with a tip resistance between 3 and 5 MΩ, which was filled with internal solution containing in mM: 130 Cs-gluconate, 8 KCl, 1 EGTA, 10 HEPES, 4 ATP, 5 QX-314; pH 7.4, 285–295 mOsm. 4–6 minutes were recorded from each cell initiated about 2-3 minutes after cell break-in. Axon patch-clamp amplifier 700B (Molecular Devices, Union City, CA) was used for voltage-clamp recordings. Cells were held at −80 mV and the recorded mEPSC data was digitized at 10 kHz by a data acquisition board (National Instruments, Austin, TX) and acquired through custom-made Igor Pro software (WaveMetrics, Lake Oswego, OR).

For intrinsic excitability measures, we performed current clamp recordings with K-gluconate internal solution (in mM: 130 mM K-gluconate, 10 mM KCl, 10 mM HEPES, 0.2 EGTA, 0.5 Na_3_GTP, 4 mM MgATP, 10 mM Na-phosphocreatine, pH 7.25, mOSM 290). Drugs were added to the ACSF to block synaptic transmission mediated by AMPA receptors (10 *μ*M 2,3-dihydroxy-6-nitro-7-sulfamoyl-benzo[f]quinoxaline-2,3-dione; NBQX), NMDAR receptors (100 *μ*M DL-2-amino-5-phosphonopentanoic acid; APV), and GABA_A_ receptors (10 *μ*M picrotoxin). Neurons were patched in current clamp and a small amount of current was injected to keep the resting membrane potential at −75 mV. Current pulses of 500 msec duration were injected at 10-second intervals with increasing amplitudes at 40 pA steps until the responses reached an asymptote (between 40 and 600 pA, or 7 steps). From the data collected, the Rheo base (minimum current needed to produce a single action potential) and average numbers of spikes per step increase in current from Rheo base were calculated for BACE KO and WT groups.

### 2.4. Data Analysis

Acquired mEPSCs were analyzed with a Mini Analysis program (Synaptosoft, Decatur, GA), with a detection threshold set at 3 times the root mean square (RMS) noise level. Recordings were excluded from analysis if the RMS noise was greater than 2, series resistance larger than 25 MΩ, and input resistance less than 100 MΩ. There was no significant difference in RMS noise across the experimental groups (Table 1). We also excluded all mEPSCs with a rise time greater than 3 ms and those that showed a negative correlation between amplitude and rise time, which may reflect dendritic filtering. 200 consecutive mEPSCs were analyzed from each cell, and the data are expressed as mean ± standard error of the mean. One-factor analysis of variance (ANOVA) followed by Newman-Keuls multiple comparison* post hoc* test was used to statistically compare data across multiple groups and Kolmogorov-Smirnov test was used for cumulative probabilities. Two-factor ANOVA was used to test interaction between genotype and the experimental variable and Student's *t*-test was used to compare measurements between WT and KO. For all tests *P* < 0.05 was considered statistically significant.

## 3. Results and Discussion

**Figure 1:**
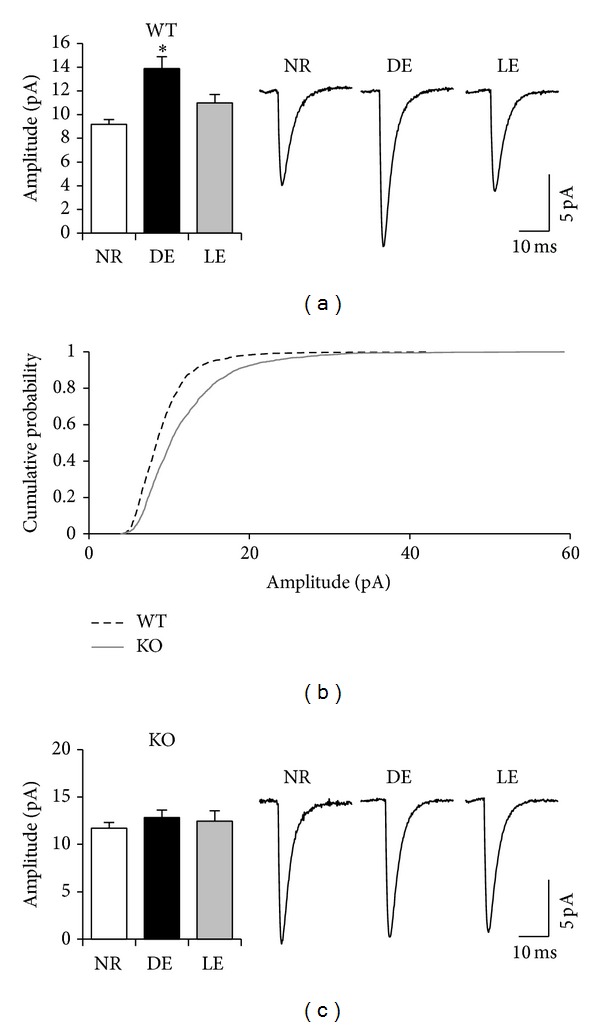
BACE1 KO mice exhibit stronger basal excitatory synaptic transmission and lack experience-dependent homeostatic regulation in superficial layers of V1. (a) In WT mice, 2 days of DE significantly increased the average amplitude of mEPSCs, which reversed to NR levels with 2 hours of LE (one-factor ANOVA: *P* < 0.001, Newman-Keuls* post hoc* test: *P* < 0.01). Left: comparison of average mEPSC amplitude. Right: average mEPSC traces from each group. (b) Cumulative probability graph comparing the mEPSC amplitude distribution of normal-reared WT (black dotted line) and KO (gray solid line). There was a statistically significant difference between WT and KO (Kolmogorov-Smirnov test: *P* < 0.0001). (c) In KO mice, there was no significant difference in the average mEPSC amplitude across groups (one-factor ANOVA: *P* > 0.39). Left: comparison of average mEPSC amplitude. Right: average mEPSC traces from each group.

**Figure 2:**
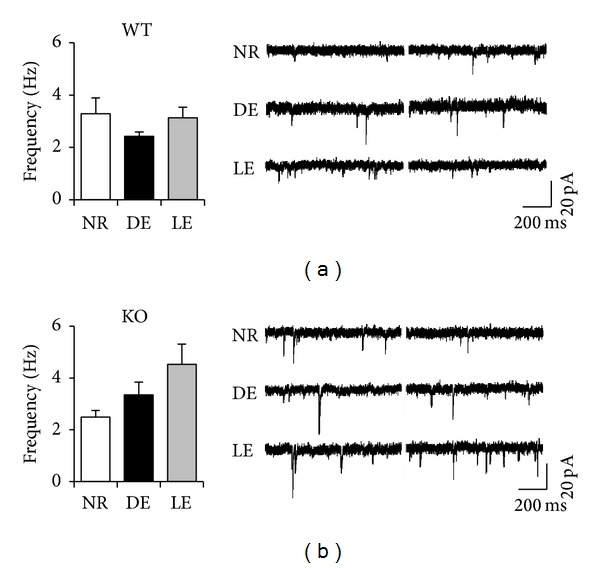
Changes in visual experience do not alter mEPSC frequency in both WT and KOs. Left: comparison of average mEPSC frequency of WT mice (a) and BACE1 KO (b). There was no significant difference across groups (one-factor ANOVA: *P* > 0.2). Right: example of raw mEPSC traces from each group.

To examine the role of BACE1 in visual cortex synaptic function and plasticity, we recorded mEPSCs in L2/3 pyramidal neurons of V1 in BACE1 WT and KO mice. To alter visual experience, mice were dark-exposed (DE) between postnatal days 22–24 (P22–24) for 2 days and a subset of them was returned to a lighted environment for 2 hours (light exposed, LE). Age-matched control mice (normal reared, NR) were kept in a normal light/dark cycle. As reported previously, in WTs 2 days of DE scaled up the amplitude of mEPSCs, which then returned to NR values after 2 hours of LE (Figure 1(a)). We found that BACE1 KO mice have significantly larger mEPSCs compared to BACE1 WTs under normal conditions (Figure 1(b)). This is consistent with a potential deficit in downscaling mechanisms in BACE1 KOs, which would result in larger basal mEPSCs. Furthermore, BACE1 KOs failed to significantly increase or decrease mEPSC amplitude with DE or LE, respectively (one-factor ANOVA: *P* = 0.4; Figure 1(c)), which suggests a lack of experience-dependent homeostatic synaptic plasticity. There was no statistically significant difference in mEPSC frequency or kinetics across genotype or experimental conditions (Figure 2, Table 1).

**Figure 3:**
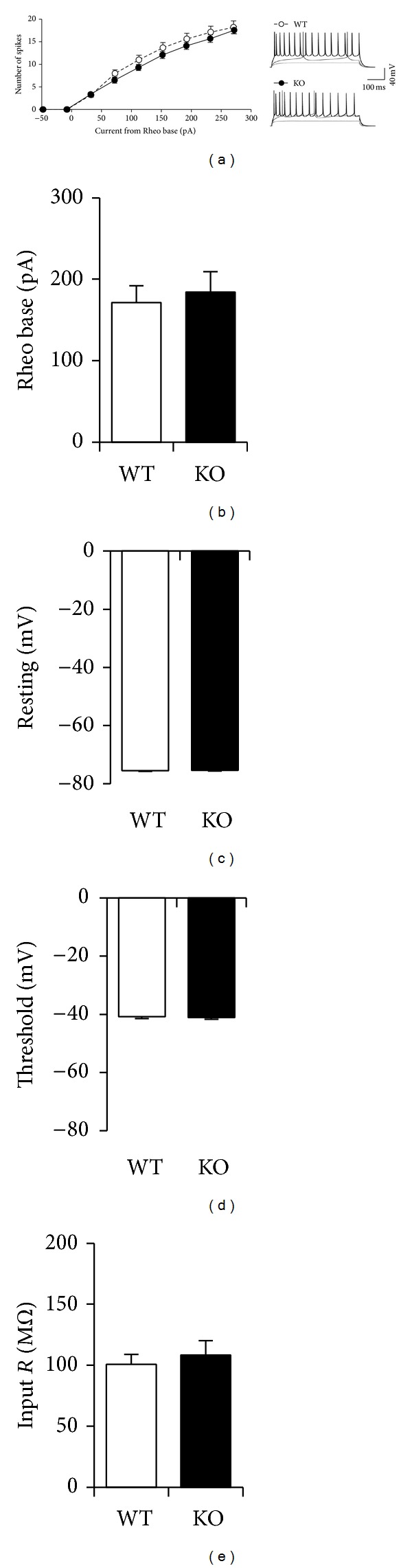
BACE1 KOs exhibit normal intrinsic excitability in L2/3 of V1. (a) Left: comparison of average action potential number with increase in current injection normalized to Rheo base (two-factor ANOVA: *P* > 0.8). *N* = 10 cells in each group (from 2 WT and 3 KO mice, 2-3 slices per mouse). Right: example of overlayed voltage traces taken at −40 pA (light gray), +40 pA (dark gray), and +120 pA (black) from Rheo base. (b–e) Comparison of average Rheo base (b), resting membrane potential (c), action potential threshold (d), and input resistance (e) measured in current clamp. Student's *t*-test: *P* > 0.6.

Previous studies showed that BACE1 KOs display heightened spontaneous seizure-like activity [40, 41] and display alterations in voltage-gated Na^+^ channel density [40, 42] (but see [41]). Therefore, the increase in basal mEPSCs of BACE1 KOs could have been due to increased spontaneous activity. However, we did not find significant difference in the intrinsic excitability of L2/3 neurons in V1 of BACE1 KOs compared to BACE1 WTs (Figure 3(a)). Furthermore, there was no difference in the Rheo base (Figure 3(b)), resting membrane potential (Figure 3(c)), action potential threshold (Figure 3(d)), or the input resistance (Figure 3(e)) measured in current clamp from neurons of BACE1 WT and KO.

Our data suggest that lacking BACE1, which abolishes A*β* production [37, 38] and several other BACE1 substrates [42–48], results in basal potentiation of excitatory synaptic transmission in L2/3 of V1 and prevents homeostatic regulation by changes in sensory experience. This phenotype mirrors that observed in Arc KOs, which we have previously reported to lack experience-dependent downscaling of mEPSCs in the same population of neurons with visual experience [29]. The larger basal mEPSC amplitude in BACE1 KOs would then reflect a lack of homeostatic downscaling with normal visual activity, and Arc KOs also exhibit a similar increase in basal mEPSC amplitude [29]. A recent study links Arc with BACE1 by placing Arc as an activity “sensor” that interacts with presenilin-1 (Psen1), which forms the active core of *γ*-secretase [49], and acts downstream of BACE1 to produce A*β* [24]. The similar phenotype seen in V1 of Arc KO and BACE1 KO further supports this idea and suggests that activity-dependent production of A*β* may play a role in homeostatic downscaling of excitatory synapses* in vivo*. This interpretation apparently contradicts a recent study using mice lacking Psen1, which cannot undergo inactivity-induced scaling up of excitatory synapses in hippocampal neurons [50]. However, this study showed that *γ*-secretase inhibitor does not block inactivity-induced scaling up of mEPSCs, which suggests that Psen1 may have an additional function in scaling up synapses separate from its role on A*β* production.

While our data are consistent with the proposed function of activity-dependent A*β* production in homeostatic control of excitatory synapses, we cannot rule out the possibility that the phenotype seen in BACE1 KO may stem from lacking other products of this enzyme. BACE1 acts on substrates other than APP, such as subunits of voltage-gated Na^+^ channels and neuregulin-1 (NRG1) (reviewed in [18]). As such, BACE1 KO mice exhibit excitability issues and defects in axon myelination [40, 47, 48]. However, the lack of a change in intrinsic excitability or spike threshold in V1 L2/3 neurons of BACE1 KO (Figure 3) suggests that altered regulation of voltage-gated Na^+^ channels [40, 41] may not be likely, at least in this brain area. Also, it suggests that the increased incidence of seizures in BACE1 KOs may reflect the increase in basal excitatory synaptic drive that we report here (Figure 1) or may be due to impaired cleaving of other BACE1 substrates such as seizure protein 6 (SEZ6) [43]. The increase in basal mEPSCs seen in BACE1 KOs may also be a result of increased APP processing through the *α*-secretase pathway in the absence of BACE1. Soluble APP*α* fragment (sAPP-*α*), which is produced by *α*-secretase cleavage of APP, has known effects on synaptic function such as facilitation of LTP [51, 52]. However, this cannot account for the lack of scaling down in BACE1 KO when reexposed to light following DE, unless facilitation of LTP would somehow counteract the effects of scaling down with increased visual activity. In addition, the locus of BACE1 expression that is critical for synaptic homeostasis is unclear and would need to be determined experimentally. It is possible that the defect in homeostatic synaptic plasticity in V1 of BACE1 KO may reflect abnormal vasculature in the retina [53]. However, this retinal phenotype was observed in older BACE1 KO; hence, it is unclear whether this phenotype is present in the younger mice used in our study or how it affects vision. Even in older BACE1 KOs there was about a 20% reduction in photopic electroretinography (ERG) without changes in scotopic ERG [53]. We have reported previously that incomplete loss of vision by bilateral eye lid suture, which reduces visually evoked responses in V1 to about 10% of normal responses [54], is ineffective at scaling up mEPSCs in the L2/3 of V1 [32]. Therefore, even with the magnitude of change in retinal function seen in older BACE1 KOs, it is unlikely that reduced visual inputs would scale up basal mEPSCs as seen in our study. A*β* has been shown to increase neuronal excitability via oxidative stress by altering intracellular Ca^2+^ dynamics (reviewed in [55, 56]). Therefore, we cannot rule out whether altered Ca^2+^ dynamics could have contributed to the BACE1 KO synaptic phenotype seen here, especially because homeostatic regulation of AMPA receptors is known to depend on changes in intracellular Ca^2+^ signals (reviewed in [57]). Furthermore, because the BACE1 KOs develop without the BACE1 gene, it is possible that the phenotype seen here may be due to compensatory changes. Hence, it is pertinent to test whether acute block of BACE1 would also result in similar synaptic deficits in future studies. Considering that even with BACE1 inhibitors, the treatment regime is likely of long duration, studying the BACE1 KO phenotype would still be of relevance. In any case, our results underscore a novel function of BACE1 enzyme in regulating experience-dependent homeostatic synaptic plasticity in neocortex. This adds to the growing list of normal physiological functions mediated by the enzyme and cautions the use of BACE1 inhibitors as AD therapeutics.

## Figures and Tables

**Table 1:** Comparison of mEPSC and neuronal parameters across experimental groups.

Genotype	Group	Frequency (Hz)	Amplitude (pA)	Rise time (ms)	Decay (*τ*, ms)	Series *R* (MΩ)	Input *R* (MΩ)	RMS Noise
WT	NR (*n* = 9;3)	3.3 ± 0.6	9.2 ± 0.4	1.7 ± 0.05	3.1 ± 0.1	23.2 ± 0.6	179 ± 18	1.6 ± 0.05
	DE (*n* = 10;4)	2.4 ± 0.2	13.9 ± 1.0*	1.6 ± 0.04	3.1 ± 0.1	20.8 ± 1.3	346 ± 83	1.7 ± 0.04
	LE (*n* = 9;3)	3.2 ± 0.4	10.4 ± 0.3	1.6 ± 0.09	3.0 ± 0.2	21.2 ± 1.6	238 ± 39	1.6 ± 0.06

KO	NR (*n* = 9;3)	2.5 ± 0.3	11.7 ± 0.6	1.5 ± 0.09	3.1 ± 0.2	22.9 ± 0.7	192 ± 36	1.6 ± 0.06
	DE (*n* = 11;4)	3.3 ± 0.5	12.8 ± 0.8	1.6 ± 0.06	3.2 ± 0.1	21.2 ± 0.9	220 ± 26	1.6 ± 0.06
	LE (*n* = 9;3)	4.5 ± 0.8	12.4 ± 1.1	1.6 ± 0.05	3.0 ± 0.3	19.4 ± 1.2	244 ± 39	1.6 ± 0.09

Values represent mean ± standard error of each measured parameter from neurons (*n*: number of neurons). *R*: resistance. *Statistically significant difference from other groups within a genotype as determined by *P* < 0.05 from one-factor ANOVA followed by Newman-Keuls Multiple Comparison *post hoc* test. *n*: number of cells; number of animals, 2-3 slices per mouse.
